# Blocking miR396 activity by overexpression *MIM396* improved switchgrass tiller number and biomass yield

**DOI:** 10.1186/s13068-024-02514-4

**Published:** 2024-05-27

**Authors:** Mingzhi Xu, Lin Li, Jianping Yan, Dayong Li, Yaling Liu, Wanjun Zhang, Yanrong Liu

**Affiliations:** 1 College of Grassland Science and Technology, China Agricultural University, Beijing, 100193 China; 2 National Engineering Research Center for Vegetables, Beijing Vegetable Research Center, Beijing Academy of Agriculture and Forestry Science, Beijing, 100097 People’s Republic of China; 3 National Center of Pratacultural Technology Innovation (Under Preparation), Hohhot, 010010 China

**Keywords:** Switchgrass, *MIM396*, miR319b, Tillering, Biomass yield

## Abstract

**Background:**

MicroRNA396 (miR396) plays an important role in the regulation of plant growth and development by repressing the expression level of its target growth-regulating factor (GRF) family genes. In our previous study, we found that overexpression of miR396 negatively regulated both tillering and biomass yield in switchgrass (*Panicum virgatum* L.). We, therefore, speculated that blocking the expression of miR396 could enhance switchgrass tillering and biomass yield. Here, we produced transgenic switchgrass plants overexpressing a target mimicry form of miR396 (*MIM396*) in wild type (WT) and *Os-MIR319b* overexpressing switchgrass plant (with higher enzymatic hydrolysis efficiency, but reduced tillering), in which the expression of miR396 was blocked. The phenotype and biological yields of these plants were analyzed.

**Results:**

Blocking miR396 to improve its target *PvGRFs* expression in switchgrass improved the tiller number and dry weight of transgenic plants. Further morphological analysis revealed that *MIM396* plants increased the number of aerial branches and basal tillers compared to those of wild-type plants. The enzymatic efficiency of *MIM396* plants was reduced; however, the total sugar production per plant was still significantly higher than that of wild-type plants due to the increase in biomass. In addition, blocking miR396 in a transgenic switchgrass plant overexpressing *Os-MIR319b* (TG21-Ms) significantly increased the *PvGRF1/3/5* expression level and tiller number and biomass yield. The miR156-target gene *PvSPL4*, playing a negative role in aerial and basal buds outgrowth, showed significant downregulated in *MIM396* and TG21-Ms. Those results indicate that miR396-*PvGRFs*, through disrupting the *PvSPL4* expression, are involved in miR319-*PvPCFs* in regulating tiller number, at least partly.

**Conclusions:**

*MIM396* could be used as a molecular tool to improving tiller number and biomass yield in switchgrass wild type and miR319b transgenic plants. This finding may be applied to other graminaceous plants to regulate plant biological yield.

**Supplementary Information:**

The online version contains supplementary material available at 10.1186/s13068-024-02514-4.

## Background

Switchgrass (*Panicum virgatum* L.) is a C4 perennial tall grass, native to North America, which has received extensive attention from researchers as a foundation plant species for ecological restoration, new energy feedstocks and also a forage crop [1]. Since 1991, it was regarded as a model bioenergy crop by the U.S. Department of Energy [2]. It has been found that improving plant morphology such as tiller number, plant height, leaf angle and lignocellulose conversion efficiency are important to increase the biomass of switchgrass. The most widely reported aspect of monocotyledon tillering is the formation of basal tillers. The novel phytohormone strigolactone (SL) has been widely reported in inhibition tiller genesis in plants [3–5]. *TEOSINTE BRANCHED1* (*TB1*) was first identified in maize [6], and its function in regulating tillering in monocotyledonous plants has also been demonstrated in wheat and rice. The mode of gibberellin (GA), a key hormone driving the Green Revolution, to regulate *MONOCULM 1* (*MOC1*) and thus increase tiller number has also been revealed [7]. In contrast to basal shoots, aerial shoots have rarely been reported. Aerial buds in monocotyledons are mostly blocking by apical dominance [8], and decreased polar auxin transport (PAT) capacity causes increased tillering in monocotyledons. From the *miR156-SPL4* module regulating aerial and basal bud sprouting, we can also speculate that other microRNAs may also have a regulatory relationship on axillary bud genesis [9]. Overexpression of rice *Osa-miR156b* gene in switchgrass resulted in a phenotype like that of overexpression of *Corngrass1* (*Cg1*), with a significant increase in tillers, plant height similar to that of the wild type, and a significant increase in biomass in the low overexpression lines [10]. In addition, miR393 in switchgrass repressed the expression of genes related to growth hormone sensitivity, such as *PvTIR1* and *PvAFB1/2/3*, which in turn led to an increase in tiller number and also biomass yield [11]. However, switchgrass has a large amount of lignin-bound cellulose and hemicellulose, and excessive lignin content reduces ethanol production in switchgrass [12]. *4-coumarate: coenzyme A ligase 1* (*Pv4CL1*) is one of the key enzymes regulating the early steps in the biosynthesis of lignin monomers, and either RNAi interference or CRISPR/Cas9 knockdown of *Pv4CL1* significantly reduced the lignin content of switchgrass and increased the enzymatic efficiency [13]. However, changing lignin content also affects plant sensitivity to biotic and abiotic stresses [14]. Therefore, it is particularly important to find relationships that can balance the relationship between biomass and sugar yield.

Plant microRNAs (miRNAs) are a class of non-coding single-stranded small molecule RNAs consisting of 20–24 nucleotides. It was reported that miRNAs play key roles in plant growth and development, response to adversity, and many other aspects by suppressing the expression levels of their target genes [15]. In recent years, more and more miRNAs have been revealed in plant development. For example, miR156 affects plant root development [16], abiotic stress [17], and tillering [18] by regulating the SPL family, miR319b regulates plant leaf development [19] and signaling [20], as well as miR397 regulates grain weight and meristem in rice [21]. MiRNA396 is one of the important miRNAs in plants, which is highly conserved in plants. MiRNA396 regulates plant growth and development through target to growth-regulating factor (GRF) family genes [22, 23]. In *Arabidopsis*, overexpression of miR396 results in reduced leaf area and cell number, a phenotype that can be restored by overexpression of *rGRF2*, which is resistant to miR396 shear [24, 25]. Meanwhile, inhibition of miR396a in Arabidopsis resulted in an increase in root length, while overexpression of miR396-shear- cleavage *AtGRF1* and *AtGRF3* significantly shortened root length [26, 27]. MiR396 is also involved in plant response to abiotic stresses, and the targeting of *NtGRF1* by *Sp-miR396a-5p* in tobacco enhanced its tolerance to salt, drought, and cold stress tolerance [28]. Transgenic plants overexpressing *Os-miR396c* in *Agrostis stolonifera* had reduced biomass of aboveground parts but enhanced their salt tolerance [29]. In a recent study, *miR396-x* and *miR396-z* were found to be significantly down-regulated in Sugarcane (*Saccharum *spp.* hybrids*) which in turn regulated sugar accumulation [30]. These reports suggest that miR396 may play a key role in regulating tillering, biomass, and sugar content in switchgrass. To date, the regulation of miR396 in plants has focused on the growth and development of leaves, flowers, and roots [31–33], and little has been reported on its regulation of aerial meristem, basal tiller development and total sugar yield in energy plants.

In our previous study, we found that overexpression of *Os-MIR319b* in switchgrass could improve salt tolerance and biomass production, but its tiller number was significantly reduced [33, 34]. In addition, we also found that miR396 has a significant positive regulatory effect on the saccharification efficiency of switchgrass cell wall, but it will reduce tillering and biomass production [35]. Based on this, we speculate that overexpressing *Os-MIR319b* and inhibiting the expression of miR396 may increase tiller number and simultaneously increase biomass yield. We first inhibited the expression of miR396 in wild-type plants and found that it can effectively increase aerial branching and basal tillering, and increase the dry matter yield of a single plant and thus the total sugar yield. Subsequent overexpression of *MIM396* in *Os-MIR319b*-overexpressing plant restored their reduced tillering phenotype. Therefore, our results indicate blocking miR396 could be used to improve biomass yield in switchgrass and may other graminaceous plants.

## Results

### Blocking miR396 increased switchgrass biomass yield

**Fig. 1:**
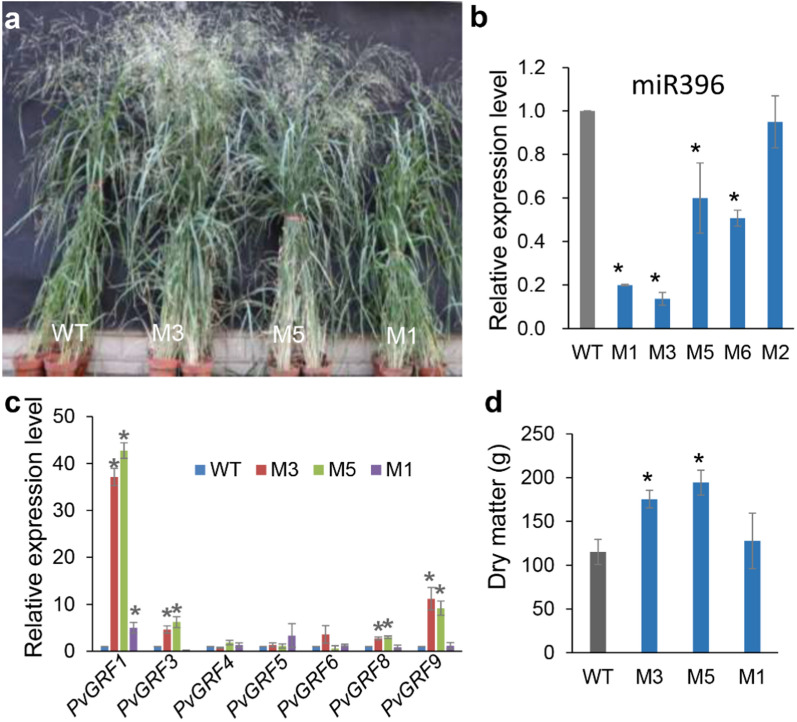
Blocking miR396 activity by overexpressing *MIM396* improved switchgrass biomass yield. **a** Typical photograph of 6-month-old wild type (WT) and *MIM396* transgenic plants (Ms). **b** The relative expression level of miR396 in WT and transgenic plants overexpressing *MIM396* (Ms). **c** The relative expression level of *PvGRFs* in WT and Ms.** d** The above-ground biomass yield of WT and Ms. Data are shown as the mean of three biological repeats ± SD. The asterisk represents a significant difference (*P* < 0.05)

We identified four *PvmiR396* genes to produce miR396 in the switchgrass genome (Fig. S1a, b, Table S1). We overexpressed the artificial gene *MIM396* to block miR396 in two callus lines (A2 and A3) of Alamo (Fig. 1a, Fig. S2a-c). After PCR identification, we obtained six *MIM396* transgenic plants (Ms), M1 to M6, regenerated from the A2 line and two transgenic plants (M8, and M9) regenerated from the A3 line (Fig. S2a). Among them, seven lines (without M4) showed RT-PCR positive (Fig. S2b). The relative expression level of miR396 in Ms (without M2) was significantly reduced compared with that in WT (regenerated wild type from A2 line) or WT3 (regenerated wild type from A3 line), respectively (Fig. 1b, Fig. S2d). And the miR396 target genes, *PvGRF1*, *PvGRF3*, *PvGRF8*, and *PvGRF9*, significantly highly expressed in M3 and M5. Only *PvGRF1* is highly expressed in M1 (Fig. 1c). In A3 line, *PvGRF1*, *PvGRF5*, *PvGRF8* and *PvGRF9* in M8 and M9 were highly expressed (Fig. S2h). For 6-month-old plants, M3 and M5 showed significant increase in biomass yield (Fig. 1d). And the significant increased biomass yield was also noticed in A3 background *MIM396* transgenic plants compared that in WT3 (Fig. S2g). Those results indicated that blocking expression of miR396 by overexpressing *MIM396* improved biomass yield in switchgrass. According to the morphological characteristics and the expression level of miR396 and *PvGRFs* in *MIM396* transgenic plants, M3 and M5 were chosen for further study.

### *MIM396* increased basal bud number and promoted aerial bud outgrowth

**Fig. 2:**
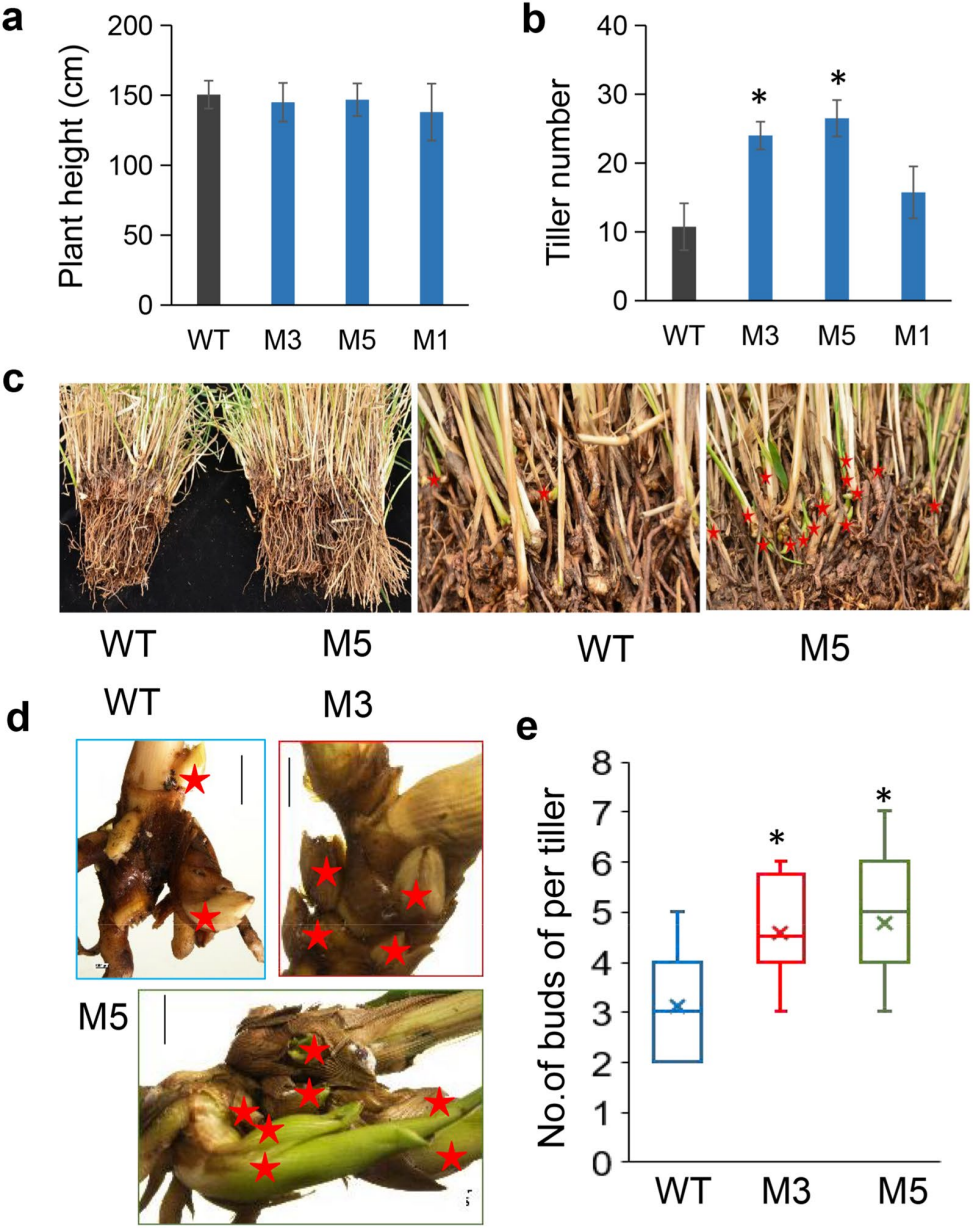
*MIM396* improved the tiller number of transgenic plants overexpressing *MIM396*. **a**, **b** Comparison of plant height (**a**) and tiller number of WT and Ms plants. **c** The photograph shows the roots of WT and M5 plants. Basal buds were marked with red stars. **d** The typical photograph of basal buds from a single tiller. Basal buds were labeled with red stars. **e** Statistical analysis of the basal bud number per tiller. (*N* = 3, *n* = 15). The data were shown as box plots with minimum to maximum plots. The center lines represent the medians, “ × ” represents the mean value, and the down and up whiskers extend to the minimum and maximum values. Plant height and tiller number data are shown as the mean of three biological repeats ± SD. The basal bud number per tiller data were assessed from three biological replicates (*N* = 3) with 15 technical repeats (*n* = 15). The asterisk represents a significant difference (*P* < 0.05)

**Fig. 3:**
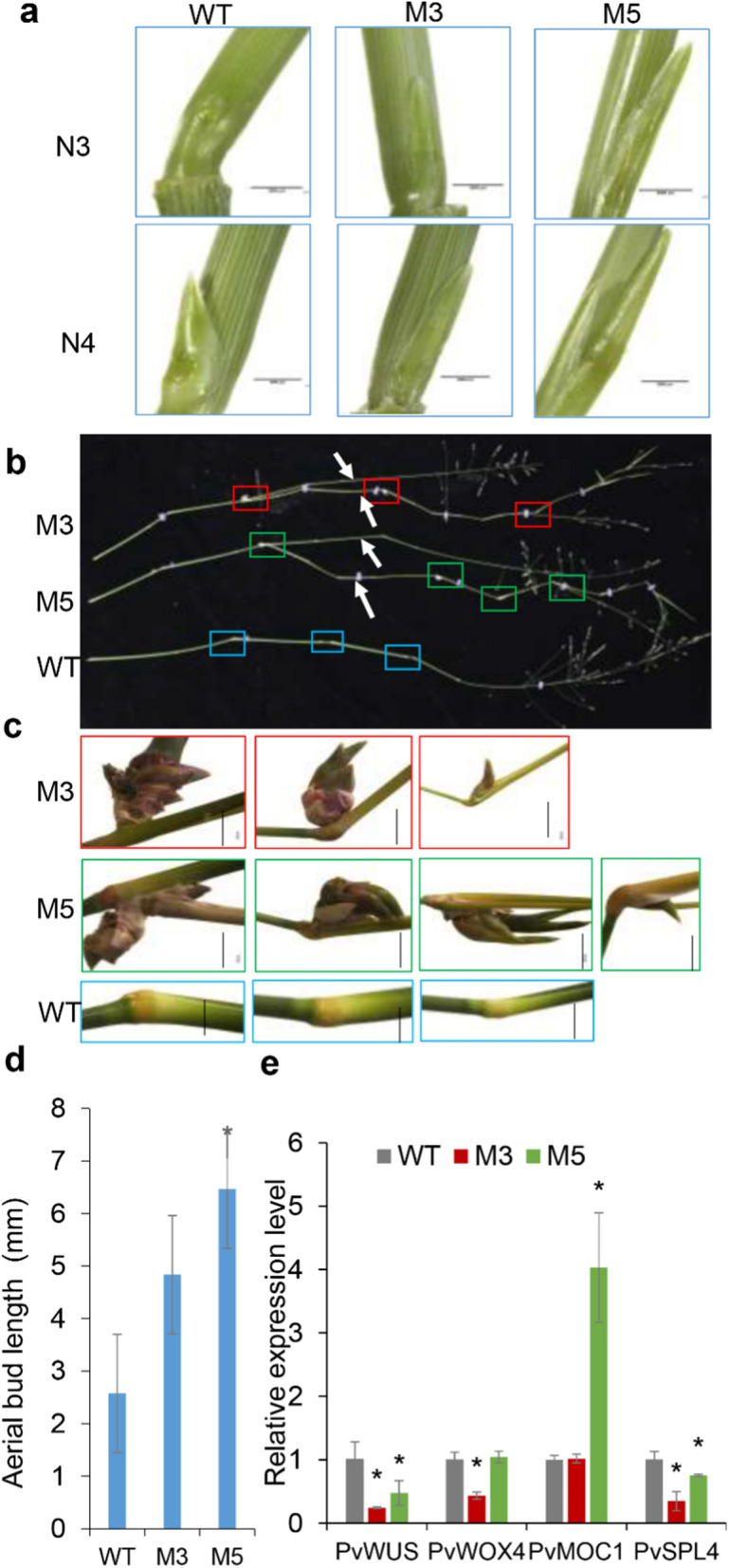
*MIM396* promotes aerial bud outgrowth. **a** The typical photograph of aerial buds of a single tiller on the stage of E4. N3 and N4 present the third and fourth internodes from the top, respectively. These photos were photographed with stereomicroscopes, and the scale bar represents 2 mm. **b** The upper stem phenotype on the R3 stage. Aerial branches were labeled with white arrows, and different color boxes marked the upper buds. **c** The typical aerial buds are photographed in figure b. The scale bar was shown as 0.5 cm. **d** Length of aerial buds in the fourth internode of the E4 stage. **e** The relative expression level associated with bud initiation and outgrowth of WT and Ms plants. The expression of WT was used as a control. Data are shown as the mean of three biological repeats ± SD. The asterisk represent significant differences (*P* < 0.05)

We carefully recorded plant phenotypes in order to investigate the mechanism of biomass improvement in Ms. As the results shown, for 6-month-old plants, there were no significant difference in plant height, leaf length and width, and stem diameter between WT and Ms (Fig. 2a, Fig. S2e, Table S2). Interesting, the tiller number of Ms showed dramatically increased by about two to three times compared that of WT (Fig. 2b, Fig. S2f). We found M5 plant contained more basal buds in root (indicate by the red star in Fig. 2c). The number of basal buds of single tiller also showed significantly increased in Ms than that in WT (Fig. 2d–e). Surprisingly, we observed the axillary buds by stereo microscopes and found the axillary buds of M3 and M5 were longer than those of WT in the third and fourth internodes on the E4 stage tiller (Fig. 3a, d), which could outgrowth of the leaf sheath as aerial buds or the secondary and third branches in Ms (indicated by the white arrows in Fig. 3b and the typical aerial buds are shown in Fig. 3c). We detected the relative expression level of bud initiation and outgrowth-related genes. The results showed that these genes were downregulated in M plants, except *PvMOC1*, which was higher in M5 (Fig. 3e). The above results indicated that miR396 promotes tillers related to the development of basal and aerial buds, which may be correlated with the expression of *PvWUS* and *PvSPL4*.

### *MIM396* improved total sugar yield due to a dramatic increase in biomass yield per plant

**Fig. 4:**
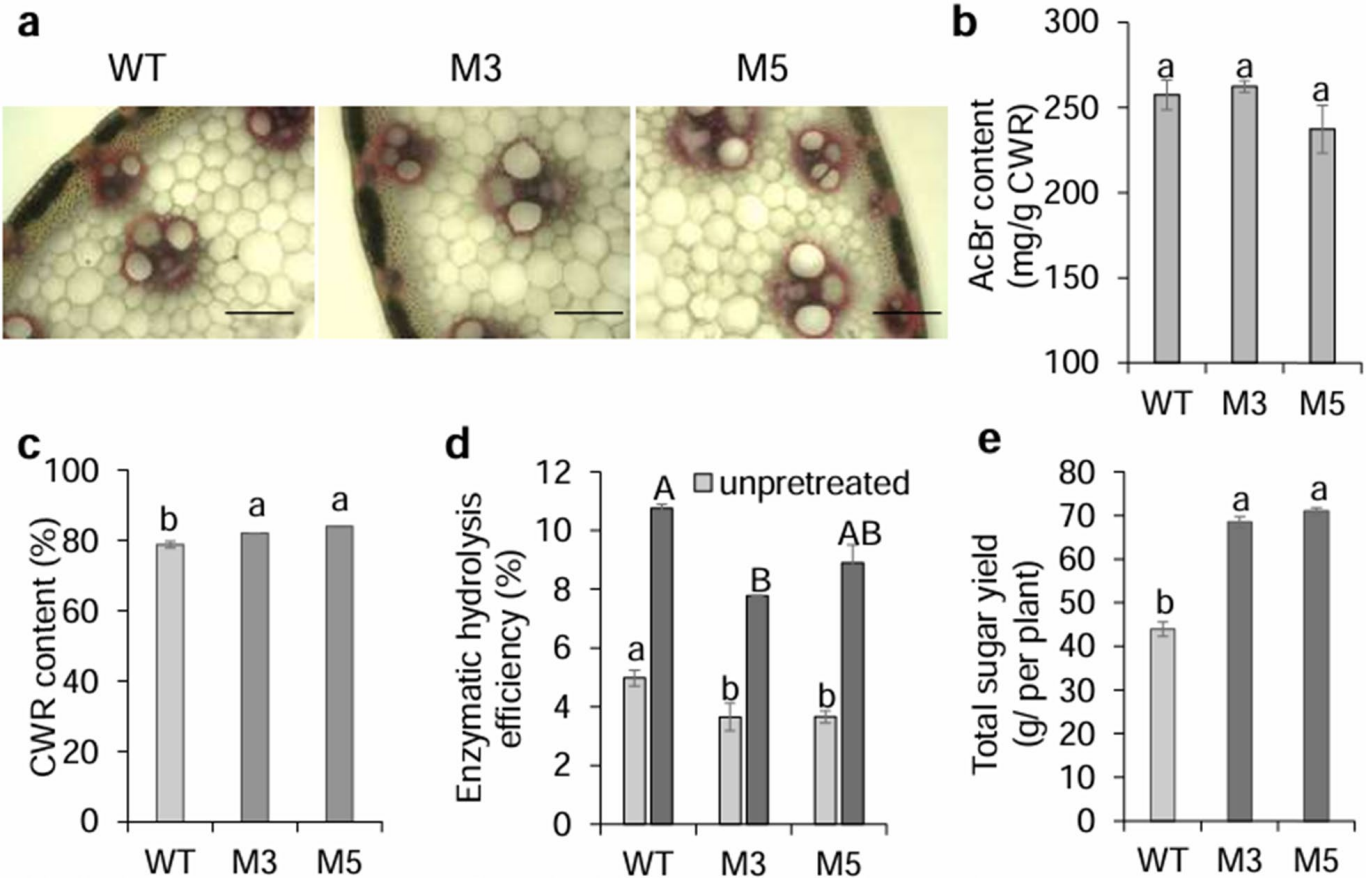
Analysis of lignin content, enzymatic hydrolysis efficiency, and soluble sugar yield. **a** Phloroglucinol-HCl staining assay of lignin content in the middle of the first internode of E3 stage cross-sections. Scale bar represents 1mm. **b** AcBr content of WT and Ms. **c** cell wall residue (CWR) content of stem. **d** Enzymatic hydrolysis efficiency with or without NaOH treatment of WT and Ms. **e** Total sugar yield per plant. Data are shown as the mean of three biological repetitions ± SD. The different letters represent a significant difference (*P* < 0.05)

In our previous report, we found miR396-*PvGRFs* negatively regulated lignin content. In this study, there were no significant difference between WT and Ms in phloroglucinol-HCl staining (Fig. 4a), AcBr lignin content (Fig. 4b) and lignin monomer content (Fig. S3). We found the cell wall residue (CWR) content of stem showed significant higher in M5 and M3 (about 82 to 84% of the dry matter) than WT (about 78% of the dry matter) (Fig. 4c). Further study showed that the enzymatic hydrolysis efficiency of Ms was significantly lower than that of WT under unpretreated and pretreated conditions (Fig. 4d). However, the total sugar yield of per plant of Ms was significantly higher than that of WT, which mainly due to higher dry matter content in Ms (Fig. 4e).

### Blocking miR396 in *Os-miR319b* transgenic plants

**Fig. 5:**
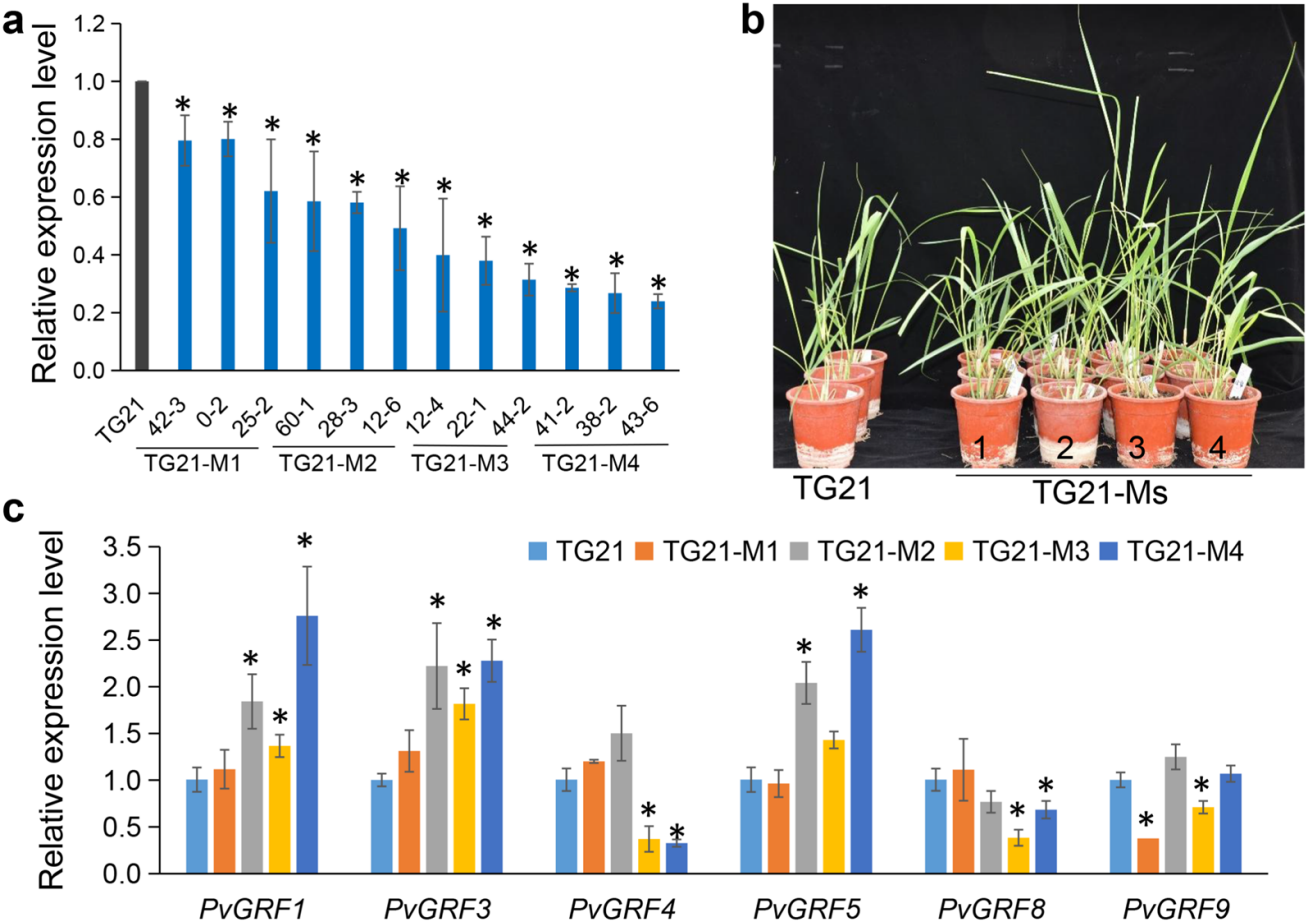
Overexpressing *MIM396* in TG21, an *Os-miR319b* transgenic line (TG21-Ms). **a** miR396 expression level in TG21 and TG21-Ms. **b** The morphological image of TG21 and TG21-Ms. **c** The relative expression level of *PvGRFs* in TG21 and TG21-Ms. Data are shown as the mean of three biological repeats ± SD. The asterisk represents a significant difference (*P* < 0.05)

In our previous report, overexpression *Os-miR319b* (TG21) significant reduced switchgrass tiller numbers [34]. To explore whether the miR396-GRF module is involved in the miR319-PCF pathway in regulating tiller number, we measured the expression pattern of miR396 precursors and *PvGRFs* in basal buds of TG21. *PvmiR396a-1* was showed up-regulation, and *PvGRF1* and *PvGRF9* showed down-regulation expression in basal buds of TG21 compared that in WT (Fig. S4a-b). In this study, we generated 12 *MIM396* PCR positive lines in TG21 (Fig. S4c). The miR396 expression level showed significant down-regulation than that in TG21 (Fig. 5a). We classified them into four groups based on the expression level of miR396 record as TG21-M1 to TG21-M4. Each group contained three transgenic lines as biological repeats (Fig. 5b). The miR396 target *PvGRFs* showed different expression pattern in TG21-Ms. *PvGRF1*, *PvGRF3* and *PvGRF5* showed up-regulation in TG21-M2, M4 than that in TG21, however, in TG21-M3, only *PvGRF1* and *PvGRF3* showed high expression (Fig. 5c).

### *MIM396* improved the tiller number of TG21

**Fig. 6:**
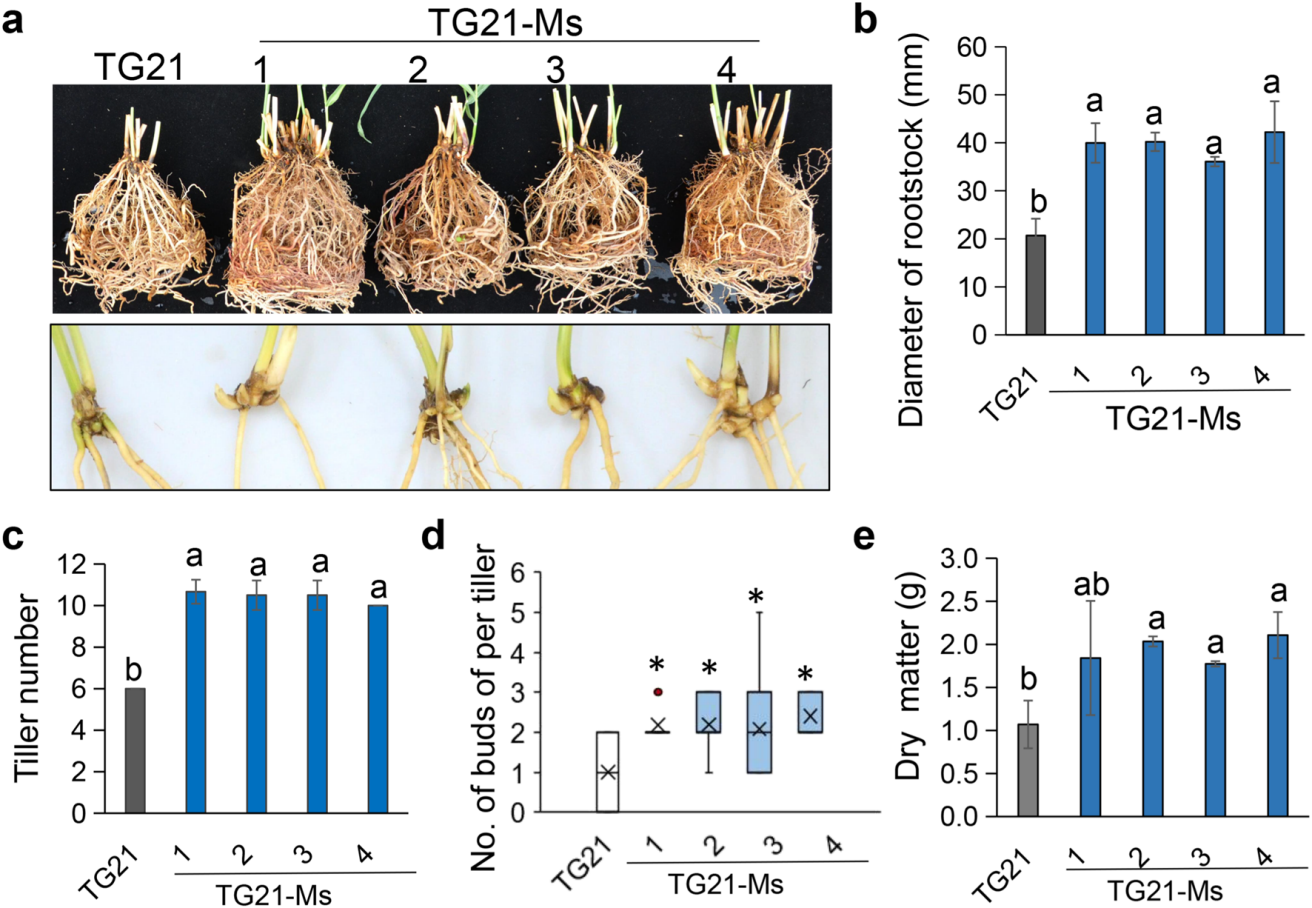
Overexpressing *MIM396* improved the TG21 tiller number and biomass yield. **a** The root phenotype of four-month-old TG21 and TG21-Ms plants and the typical photograph of basal buds of single tiller. **b** Analysis statistic of diameter of rootstock. **c** Tiller number per plant. **d** Number of buds per tiller. At least 15 numbers were used for measurement. The data were shown as box plots with minimum to maximum plots. The center lines represent the medians, “ × ” represents the mean value, and the down and up whiskers extend to the minimum and maximum values. The asterisk represents a significant difference (*P* < 0.05). **e** Above ground biomass yield per plant. Data was shown as the mean of three biological repeats ± SD (**c**, **d** and **e**). The different letters represent a significant difference (*P* < 0.05)

Firstly, we stained the DMS powder of TG21-Ms and found that *MIM396* did not have a significant effect on lignin content in the TG21 background (Fig. S4d). Therefore, we subsequently focused mainly on its effect on tillering and dry weight. As Fig. 6a shown, the root of TG21-Ms was significant bigger than TG21, and the diameter of rootstock significant thicker than TG21 (Fig. 6b). Compared with TG21, tiller number of per plant of TG21-Ms was significant increased than TG21 (Fig. 6c). Analysis the buds number per tiller also showed that the TG21-Ms had more buds than that of TG21 (Fig. 6d). Obviously, those phenotypes resulted in a significant higher dry biomass yield of TG21-Ms than TG21 (Fig. 6e). We also examined the expression of *PvWUS*, *PvWOX4*, *PvMOC1* and *PvSPL4* in TG21-M1 to TG21-M4, and we found that *PvSPL4* appeared to be down-regulated compared to TG21, but *PvWUS*, *PvWOX4* and *PvMOC1* do not show a trend exactly in line with *MIM396*. (Fig. S4e).

## Discussion

The plant architecture and sugar yield of switchgrass is important for improving its energy production. MiR396 plays an important role in plant leaf, flower, and root growth and development, such as negatively regulating leaf area, leading to inflorescence anomalies as well as increased root length [24, 36, 37]. We had demonstrated that the miR396-GRF module negatively regulated plant height and biomass yield in switchgrass [35]. And, highly overexpressed miR396 lines showed a reduction in tiller number [35]. In this study, our data clearly showed that blocking miR396 by overexpressing *MIM396* resulted in a dramatic increase in tiller number and dry weight but did not effect the plant height in switchgrass. Several reports have shown similar phenomena, such as overexpression of miR319b, which significant reduced tiller number, but *MIM319* transgenic plants did not increase it [38]. Those contradictions were boldly speculated to be resulting from the not quite opposite expression pattern of miRNA-target genes in miRNA overexpression and inhibiting transgenic plants. We found all miR396-target *PvGRFs* were downregulated in overexpression miR396 transgenic plants [35], but only *PvGRF1/3/5/8/9* (without *PvGRF4*) showed upregulation in *MIM396* transgenic plants. Our previous report showed that different *PvGRFs* hold specific and redundant functions in switchgrass. Such as *PvGRF1* and *PvGRF9*, but not *PvGRF3*, play positive roles in regulating switchgrass plant height but have no significant effect on tiller number [38]. In this study, the *MIM396* transgenic line M1, which upregulated *PvGRF1* expression only, showed no significant difference in tiller number compared with WT. In the transgenic plants of TG21-Ms only *PvGRF1*, *PvGRF3*, and *PvGRF5* showed up-regulated expression. However, PvGRF3 and PvGRF5 showed different expression patterns in MIM396 transgenic plants from A2 and A3 backgrounds. We boldly speculate that *PvGRF3* and *PvGRF5* may play a key and redundancy role in improving switchgrass tiller number, which is worthy for further study.

The site of tiller development in grass plants determines plant architecture, and in monocotyledonous plants there are two types of tillers, the aerial and basal buds. Many genes regulating basal bud genesis have been identified [39–42], in contrast to aerial buds, which develop from stem nodes and require different nutrients for growth than basal buds, which are entirely transported by the main stem. There are limited studies on aerial buds, and the miR156-*SPL4* module has been reported to regulate aerial bud and basal bud development [9]. In addition, genes that regulate the formation of axillary bud meristematic tissue such as *SHOOT MERISTEMLESS* (*STM*) and *WUSCHEL* (*WUS*) also play a key role in aerial bud genesis [43, 44]. In this study, miR396 was found to affect the aerial bud development. As aerial buds developed to a later stage, inhibition of miR396 expression significantly promoted the growth of aerial buds. Meanwhile some genes regulate bud growth such as *WUS*, *WOX4*, *MOC1*, and *SPL4* responded differently, especially the down-regulated expression of *PvSPL4* in the MIM396 transgenic material is consistent with the previously reported results of negative regulation of aerial buds [9]. MiR396 has a highly conserved sequence in plant. Blocking the expression of miR396 has been shown to reduce tillering in *Agrostis stolonifera* [29].We found that the number of basal shoots was significantly increased in the *MIM396* plants. These results suggest that the effect of miR396 on tillering is mainly related to the development of basal buds.

Biomass yield and feed stock quality are vital to energy plants. In switchgrass, cellulose, hemicellulose, pectin, and lignin are the main components that make up the cell wall. As lignin is a key factor affecting the utilization efficiency of forage, changing the lignin composition, or reducing the lignin content to improve switchgrass and enhance its digestibility and utilization has received more and more attention. Generally, increasing biomass yield while decreasing lignin content in switchgrass is important breeding goal. *Pv4CL1*, *PvCOMT* and *PvF5H* play important roles in lignin content [13, 45, 46], such as the down-regulation of *PvF5H* in transgenic switchgrass that inhibits *PvCOMT* further hinders the biosynthesis of S-lignin monomers, thus increasing the biosynthesis of monomer biosynthesis, thereby increasing G-lignin monomer biosynthesis. In this report, we found that the CWR of *MIM396* transgenic plants was significantly higher than that of WT. And the enzymatic efficiency of both treated and untreated *MIM396* was significantly lower than that of the wild type. However, interestingly, when we evaluated the final sugar yield, we found that the total sugar yield of *MIM396* was significantly higher than that of the wild type in a single plant, which mainly due to the dramatical increase in dry matter yield. It also suggests that miR396 has a strong correlation between the regulation of biomass quality and yield.

MiR319b is also highly conserved across species and also plays a key role in regulating plant architecture. However, miR319 does not have the same function in regulating tillering in different species, for example, it inhibits tillering in rice but exhibits a promoting effect in wheat [39, 47]. In our previous study, we found that heterologous expression of *Os-miR319b* increased the leaf width and leaf thickness, promoted switchgrass stem elongation, and increased stem diameter, but reduced the number of tillers [38]. It has been reported that miR319-target TCPs activate the expression of miR396 in *Arabidopsis* [22]. However, in this study, we found miR396 upregulated and *PvGRFs* downregulated in *Os-miR319b* transgenic plants, which indicates the specific regulating mechanism between miR319-TCP and miR396-GRF in different varieties*.* We therefore created transgenic switchgrass (TG21-Ms) overexpressing both *Os-miR319b* and *MIM396* and found that the expression pattern of *GRF* was also altered in the transgenic plants. Subsequently, our morphological analysis of TG21-Ms and TG21showed that MIM396 significant increased tiller number and dry biomass yield in TG21. Those results suggested that miR319-TCP module reduced switchgrass tiller number by increasing miR396-PvGRF module, at least partly. Interestingly, miR156-target gene *PvSPL4* was down-regulated in both *MIM396* transgenic plants and TG21-Ms lines. It has been reported that miR156-target *PvSPL4* negatively regulates the development of aerial and basal tillers in switchgrass [9]. This predicts that the miR396-GRFs module may be epistatic for the miR156-SPLs module in regulating the tiller number and yield of switchgrass. In a word, we seemingly explored an interesting and valuable pathway about miR319-PvPCFs, miR396-PvGRFs, and miR156-PvSPLs in regulating switchgrass biomass yield and lignin content, which is much more worthy of in-depth studies to establish this relationship.

## Conclusions

In this study, we created transgenic materials that suppressed miR396 expression to improving *PvGRFs* expression in switchgrass and analyzed their plant height, tiller, dry weight, and total sugar yield, and found that they were significantly increased compared with the wild type except for plant height, which may be due to repressing the miR156-target gene *PvSPL4* expression. This is the first report that miR396 regulates both aerial and basal buds in monocotyledonous plants, which enriches the research on aerial buds in monocotyledonous leaves. In addition, suppression of miR396 expression in switchgrass overexpressing *Os-miR319b* could complement the phenotype of its reduced tillering. The results showed that inhibition of miR396 significantly increased the biological yield as well as the number of tillers in switchgrass, which provides a new method to improve the biomass yield of C4 energy plants and may also other grasses.

## Materials and methods

### Plant materials

Due to the complexity of the genetic background of switchgrass, to ensure the consistency and rigor of the experiments, the transgenic plants and wild type were obtained from one highly regeneratable callus line. In this study, we generated two highly regeneratable callus lines (A2 and A3) from two Alamo seeds. To generate callus, the juvenile spike of the *Os-miR319b* transgenic line TG21 was used. According to our earlier studies, those calluses lines were used as explants to produce *MIM396* transgenic plants, respectively. The recombinant vector of overexpression *MIM396* gene was reported in our previously reported method [48]. These transgenic plants were grown in a greenhouse with 16 h light/8 h dark at 25 °C.

### PCR and qRT-PCR analysis

Plant leaf DNA was isolated and used as a template for PCR testing. Total RNA was extracted using Trizol regent. Then, one μg RNA after eliminating genomic DNA was used to reverse transcription into the first strand of cDNA using the random primer oligo (dT) or stem-loop RT primer (for miR396) (Takara, Dalian, China RR047A). The cDNA was used as template for qRT-PCR analysis using the kit (Beijing Qihengxing Biotechnology Co., LTD, FS-Q1002 kit). Data were collected with a qTOWER3G (analytik jena). The switchgrass ubiquitin gene (AP13CTG25905) or the nuclear small RNA U6 cDNA (Pavir.J34795.1) (for miR396) was used as internal control. The relative expression level were calculated using the 2^−∆∆CT^ measurement method [34]. Three biological repeats were performed and used for statistical analysis. The primers used in this study were listed in Table S3. All primers avoided the miR396 shear site.

### Plant growth conditions and phenotype analysis

Plants were cultured in a greenhouse and photoperiod was adjusted to 14 h/10 h (day/night) for four months. Each *MIM396* line were propagated to four plants by tillers as biological replicates. After 6 months of growth, at least 20 R3 stage tillers (fully emerged spikelets and peduncle) of each plant (as technical replicates) were used to collect data of morphology indexes. Two-month-old TG21-Ms plants were divided into four groups according to the miR396 expression level. Each group contained three lines as biological duplicates for morphology indexes. The plant morphology indexes including tiller number, plant height, stem diameter, leaf length and width were measured and statistic analysis following the method of our previous report [38]. The aboveground biomass of each plant was harvested, dried at 65 ℃ for 3 days, then weighted separately. The number of basal buds of single tiller from 15 tillers were counted. The diameter of junction between tiller and root were measured. The typical photograph of aerial buds and basal buds were captured using Stereo Microscope (Nikon, C-DSS230).

### Determination of AcBr lignin content and enzymatic hydrolysis efficiency

The switchgrass plant at R3 stage were harvested, dried and sieved through 1 mm sieve for extracting cell wall residues (CWR) as described by Chen and Dixon [49]. Measurement of AcBr lignin content, enzymatic hydrolysis assay, and the solubility sugar yield were performed using our previously reported methods [35]. In brief: for AcBr ligin content, CWR was weighed into a reaction bottle and were immersed in 5 mL acetyl bromide reagent (25%, *v*/*v*) and heated to 50 ℃. After 4h, we transferred the supernatant (about 4 mL) into a 50 mL volumetric flask, to which 10 mL of 2M NaOH, 12 mL of acetic acid and 1 mL of 0.5 M hydroxylamine were added followed by the addition of glacial acetic acid to bring the total volume to 50 mL. The absorbance was measured at 280 nm by a spectrophotometer. For enzymatic hydrolysis assay, CWR pretreated or unpretreated with 0.25 M NaOH and digested by exposing to 0.5 mL enzyme mixture and treated with the same amount enzyme by following mentioned our previous report. The percentage of released carbohydrate content after enzymatic hydrolysis to the total carbohydrate content as enzymatic hydrolysis efficiency. The soluble sugar yield of per plant was calculated using total carbohydrate content of each plant and enzymatic hydrolysis efficiency.

The solubility sugar yield of per plant = the per plant biomass (g) × CWR content × cell wall carbohydrate yield (g/g CWR) × enzymatic hydrolysis efficiency.

### Histological analysis

The hand-cut section and dry matter of stem powder were stained with phloroglucinol/HCl to asses lignin content as our previous reported method [35].

### Statistical analysis

In the experiment, data from biological repeats were analyzed by one-way ANOVA through the proc GLM for ANOVA of SAS 8.2 (SAS Institute, Cary, NC, USA). The comparison of treatments was separated by Duncan’s multiple range test (*P* < 0.05).

### Supplementary Information

Supplementary Material 1.

## Data Availability

All data generated or analyzed in the present study are included in this article and in additional information.
