# Supplementary material for: Blocking miR396 activity by overexpression MIM396 improved switchgrass tiller number and biomass yield
Source: Biotechnol Biofuels Bioprod. 2024 May 27;17:69. doi: 10.1186/s13068-024-02514-4 (PMC11131217; doi:10.1186/s13068-024-02514-4)
Supplement: Supplementary file 1 — Supplementary Material 1. [file 13068_2024_2514_MOESM1_ESM.docx]

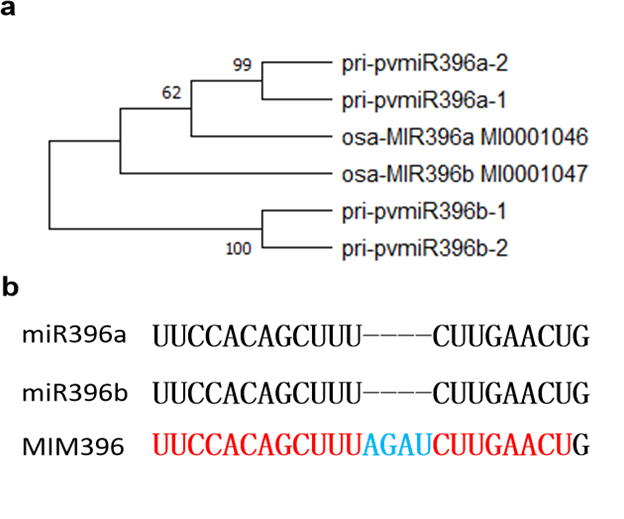
**Fig. S1 a** Phylogenetic analysis of miR396 precursors from *Oryza sativa* (osa) and *Panicum virgatum* (pv). **b** The sequences of mature miR396 and the artificial interference sequence of MIM396.


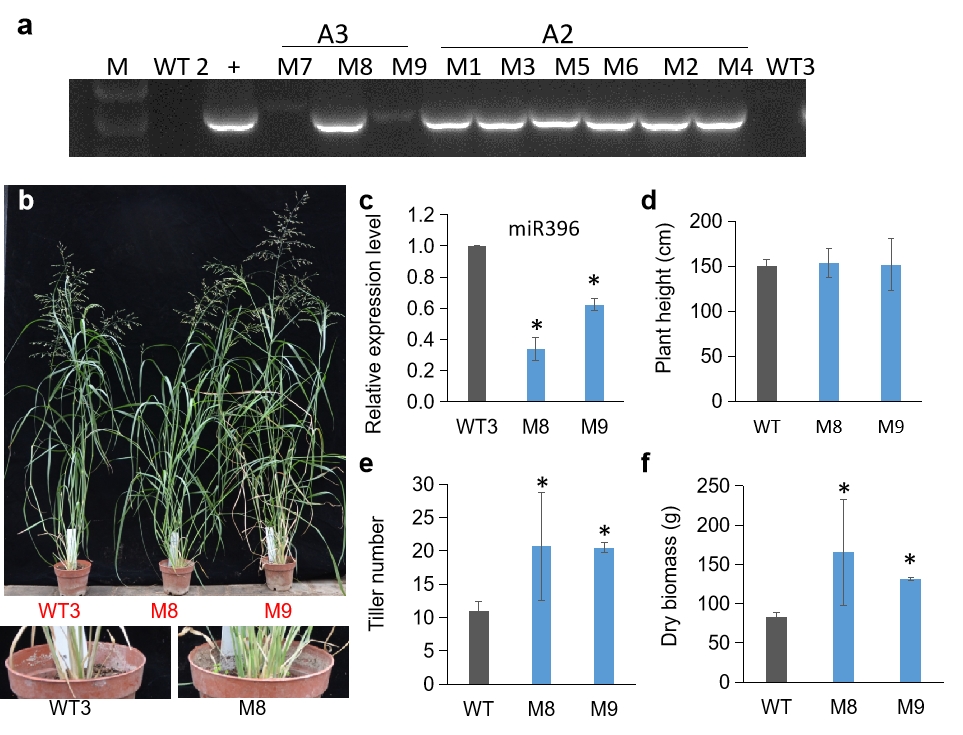


**Fig. S2** The PCR analysis and morphological characteristics of WT3 and *MIM396* transgenic plants (M8 and M9) regenerated from the same calluse line. (**a**) The PCR analysis of MIM396 transgenic plants (**b)** The typical photograph of four-month-old WT3 and Ms. **(c)** The relative expression level of miR396 in WT3 and Ms. **d-f** Statistical analysis of plant height (**d**), tiller number (**e**), and above-ground dry matter (**f**). Data are shown as the mean of four biological repeats ± SD. The asterisk represents a significant difference (*P* < 0.05).


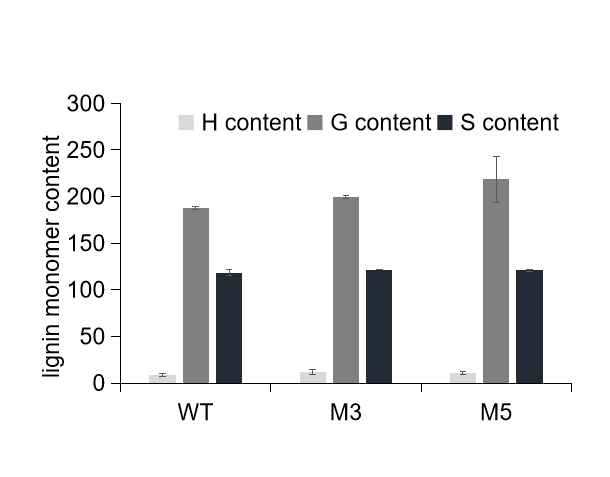


**Fig. S3** Comparison of lignin monomers *p*-hydroxyphenyl (H), syringyl (S), and guaiacyl (G) content in WT and Ms. Data are shown as the mean of three biological repeats ± SD.


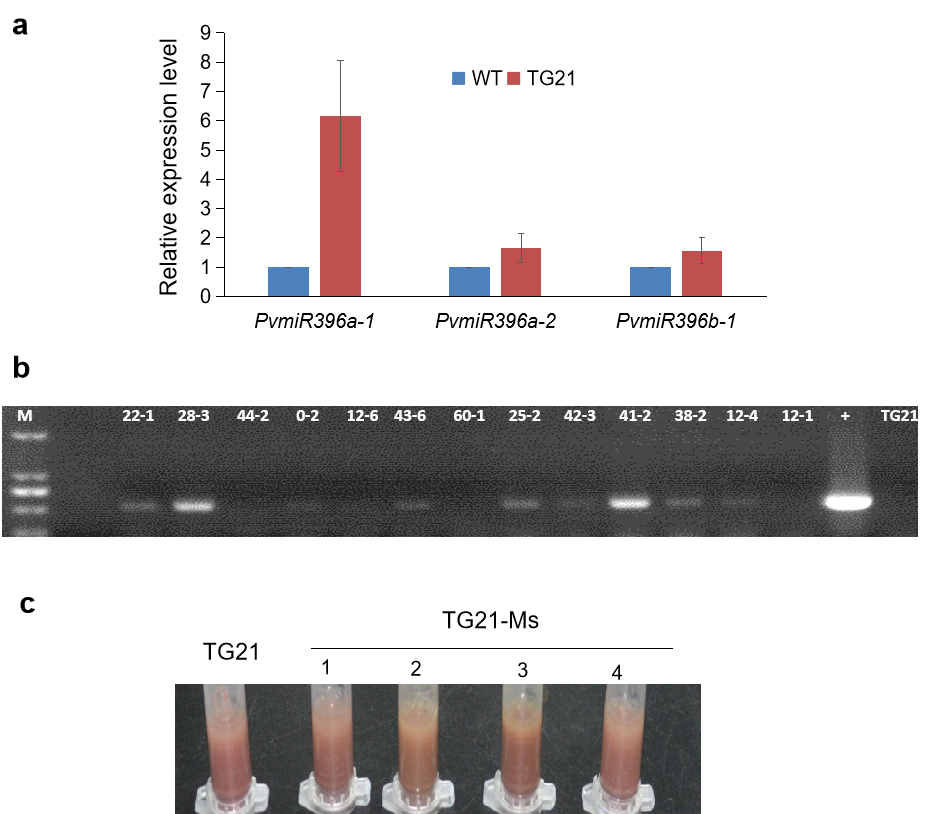


**Fig. S4** PvmiR396 genes expression pattern in WT and TG21. (**a)** The qRT-PCR analysis of miR396 expression in TG21. (**b**) PCR analysis of MIM396 transgenic plants in the TG21 background. (**c**) Phloroglucinol–HCl staining assay of lignin content in the TG21-M1, 2, 3, and 4 dry materials powder of stems. Data are shown as the mean of three biological repeats ± SD.

Table S1 Morphological characteristics of WT and Ms

| Lines | Leaf length (cm) | Leaf blade width (cm) | Stem diameter (mm) |
| --- | --- | --- | --- |
| WT | 56.00±7.46 | 10.17±0.46a | 2.99±0.35 |
| M3 | 41.78±10.29 | 9.28±0.80 ab | 2.49±0.37 |
| M5 | 48.50±2.83 | 8.47±0.72 b | 2.30±0.06 |
| M1 | 43.93±4.96 | 8.80±1.05 | 2.20±0.33 |
| WT3 | 54.33±8.00 | 10.47±0.89 | 3.17±0.25 |
| M8 | 54.02±3.46 | 9.59±0.70 | 2.66±0.14 |
| M9 | 57.25±3.80 | 11.95±1.06 | 2.81±0.33 |

Table S2 The primers used in this study

| Primer name | Sequence(5’-3’) |
| --- | --- |
| PCR |  |
| MIM396-F | AAGAAAAATGGCCATCCCCTAGC |
| MIM396-R | GAGGAATTCACTATAAAGAGAATCG |
| Reverse transcription reaction |  |
| stem-loop miR396a | GTCGTATCCAGTGCAGGGTCCGAGGTATTCGCACTGGATACGACCAGTTC |
| qRT-PCR | |
| pri-pvmiR396a-1-F | TCTTCAGCCGCTTGCAGACG |
| pri-pvmiR396a-1-R | TGCCTATCACCGAATTGGAGAGAC |
| pri-pvmiR396a-2-F | GGGAAGGTATACGCACCGATAGAG |
| pri-pvmiR396a-2-R | ATCAAAGCATGGAGGAAAGCAAGC |
| pri-pvmiR396b-1-F | TTGGGTCCCCGTCATCTCTCTC |
| pri-pvmiR396b-1-R | TTCACGCAGCAGTTCAAGAAAGC |
| F_PvUBIQUITIN | CAGCGAGGGCTCAATAATTCCA |
| R_PvUBIQUITIN | TCTGGCGGACTACAATATCCA |
| 396a-Forward | CGGCGGTTCCACAGCTTTCTT |
| 396a-Reverse | GTGCAGGGTCCGAGGT |
| PvGRF1-F | CGCACGAGAAATCAAACAAGG |
| PvGRF1-R | CATCATTACGGTAGCGGGAG |
| PvGRF2-F | GTCCTCCTCTATCCTCCGTTGG |
| PvGRF2-R | TGCCCGCTCAGCATCATCTC |
| PvGRF3-F | ATGAGGTCAGCACCAGAGAGTCGT |
| PvGRF3-R | CGAGCGGGAATACCATCAAG |
| PvGRF4-F | AAAACTCCGCATTTCCTCTCTC |
| PvGRF4-R | AGGGGCTGCCTGTCCATCTT |
| PvGRF5-F | CCCCGAACCTATTTCTTTCACTG |
| PvGRF5-R | GCACACAAACACTTGCATCATC |
| PvGRF6-F | CAAGAACGGGTAACGGCGAAG |
| PvGRF6-R | GGATGCTCGGATGGTTTACTACA |
| PvGRF8-F | GCCGAGATGGGTTCTTTGG |
| PvGRF8-R | ACTGCGTCGGAGTGAATGG |
| PvGRF9-F | CAGAACCTGGAAGATGCCGT |
| PvGRF9-R | GCTTTCTTGAACGATGACGATT |
| PvWUS-F | CATCGAGACATGCGTCCTG |
| PvWUS-R | GGGAAGAGAGGGAGCGTCT |
| PvWOX4-F | TGCAGCTGAAACCAAAGATG |
| PvWOX4-R | AGTTCCAGGGTCACATCGTC |
| PvMOC1-F | GTGTCGTCGTGGCAGTAGC |
| PvMOC1-R | TTGCAACAAAATGCAATGCT |
| PvSPL4-F1 | ACTGCCGCCACAAGGTGT |
| PvSPL4-R1 | GGTGGAACCTGCTGCACT |
